# Comparison of bone and articular cartilage changes in osteoarthritis: a micro-computed tomography and histological study of surgically and chemically induced osteoarthritic rabbit models

**DOI:** 10.1186/s13018-021-02781-z

**Published:** 2021-11-08

**Authors:** Sharifah Zakiah Syed Sulaiman, Wei Miao Tan, Rozanaliza Radzi, Intan Nur Fatiha Shafie, Mokrish Ajat, Rozaihan Mansor, Suhaila Mohamed, Angela Min Hwei Ng, Seng Fong Lau

**Affiliations:** 1 Department of Veterinary Clinical Studies, Faculty of Veterinary Medicine, Universiti Putra Malaysia, 43400 UPM Serdang, Selangor Malaysia; 2 Department of Veterinary Preclinical Studies, Faculty of Veterinary Medicine, Universiti Putra Malaysia, 43400 UPM Serdang, Selangor Malaysia; 3 Department of Farm and Exotic Animals Medicine and Surgery, Faculty of Veterinary Medicine, Universiti Putra Malaysia, 43400 UPM Serdang, Selangor Malaysia; 4 UPM-Makna Cancer Research Laboratory (CANRES), Institute of Bioscience, Universiti Putra Malaysia, 43400 UPM Serdang, Selangor Malaysia; 5 Tissue Engineering Centre, Universiti Kebangsaan Malaysia Medical Centre, 56000 Cheras, Kuala Lumpur, Malaysia

**Keywords:** Osteoarthritis (OA), Subchondral bone, Articular cartilage, Micro-computed tomography (micro-CT), Histology, Anterior cruciate ligament transection (ACLT), Monosodium iodoacetate (MIA)

## Abstract

**Background:**

Osteoarthritis (OA) is a multifaceted condition that affects both the subchondral bones and the articular cartilage. Animal models are widely used as an effective supplement and simulation for human OA studies in investigating disease mechanisms and pathophysiology. This study is aimed to evaluate the temporal changes of bone and cartilage in surgically and chemically induced osteoarthritis using micro-computed tomography and histology.

**Methods:**

Thirty rabbits underwent either anterior cruciate ligament transection (ACLT) procedure or injected intraarticularly with monosodium iodoacetate (MIA, 8 mg) at the right knee joint. The subchondral bones were scanned via micro-CT, and articular cartilage was assessed histologically at 4-, 8- and 12-week post-induction.

**Results:**

Based on bone micro-architecture parameters, the surgically induced group revealed bone remodelling processes, indicated by increase bone volume, thickening of trabeculae, reduced trabecular separation and reduced porosity. On the other hand, the chemically induced group showed active bone resorption processes depicted by decrease bone volume, thinning of trabeculae, increased separation of trabecular and increased porosity consistently until week 12. Histologically, the chemically induced group showed more severe articular cartilage damage compared to the surgically induced group.

**Conclusions:**

It can be concluded that in the ACLT group, subchondral bone remodelling precedes articular cartilage damage and vice versa in the MIA group. The findings revealed distinct pathogenic pathways for both induction methods, providing insight into tailored therapeutic strategies, as well as disease progression and treatment outcomes monitoring.

**Supplementary Information:**

The online version contains supplementary material available at 10.1186/s13018-021-02781-z.

## Background

Osteoarthritis (OA) is characterised by degeneration of the articular cartilage, changes in the subchondral bone, and inflammation of the synovium [1]. Osteoarthritis was previously thought to be a disease affecting only articular cartilage [2, 3], but recent findings suggested that subchondral bones are also importantly involved in OA pathogenesis [4, 5] and there is much debate in the literature on the cartilage–bone interaction. Subchondral bones and articular cartilage are complementarily involved in biomechanical load-bearing joints [1].

Osteoarthritis is characterised by primary OA (idiopathic), which is due to degenerative changes at the joint. This condition is mainly attributed to ageing, while secondary OA is associated with factors such as obesity, joint injury, trauma and congenital disease [6]. In understanding this disease, animal models serve as an important complement and simulation for human OA studies. Animal models are classified into two types: spontaneous and induced models. Spontaneous models consist of naturally occurring and genetic models [7]. Induced OA models are divided into surgical and chemical models.

Surgical induction such as anterior cruciate ligament transection (ACLT) causes destabilisation of the joints leading to post-traumatic osteoarthritis (PTOA) [8] and mimics articular cartilage degeneration after ACL rupture. Chemical models are developed by injecting altering factors into the joints or administering noxious agents systemically. In chemically induced models, monosodium iodoacetate (MIA) is the most commonly used compound in OA study [9]. Monosodium iodoacetate will inhibit glyceraldehyde-3-phosphate dehydrogenase of the Krebs cycle that is responsible for the death of chondrocytes. This event culminates in the formation of osteophyte and degradation of articular cartilage [10]. Intraarticular injection of MIA leads to decreased number of chondrocytes and caused histological and morphological changes of the articular cartilage that were similar to changes in human degenerative OA [11]. While both induction methods are proved to successfully develop OA, the pathogeneses and progression are different. Chemically induced model is less invasive, and no surgical procedure is needed, and therefore, reducing the risk of possible infection [7]. This model has slower progress compared to surgically induced model as it showed inflammatory change in the early stages of OA [8]. On the other hand, surgical models produce highly reproducible results and rapid disease progression and therefore it is the best choice for short duration studies. However, this form of invasive and rapid induction is too quick to show early stages of OA development or to measure early drug treatments [12]. The difference in time-dependent progression between both induction methods is suggested to be caused by different pathogeneses between two types of OA.

Typically, most OA studies focused solely on the changes in articular cartilage [13, 14]. There is a data paucity regarding the evaluation of changes in articular cartilage and subchondral bones for different induction methods at different times. Previous studies have shown that intraarticular injection of MIA has distinctive pathophysiology, with no correlation with post-traumatic OA represented by ACLT method [15]. Therefore, this study elaborates on the onset of OA; whether it is cartilage or bone driven, and temporal changes of both cartilage and bone in different induction methods. This information is important in understanding the pathophysiology of the disease and subsequently planning targeted therapeutic strategies and specifically monitoring disease and treatment outcome.

## Materials and method

### Animals

A total of 35 male New Zealand white rabbits were used in this experimental study. The rabbits were obtained from A-Sapphire Enterprise, Malaysia. The animals were between eight and nine months old, weighing 1.8–2.0 kg, and were placed in the Animal Research Facility, Faculty of Veterinary Medicine, Universiti Putra Malaysia. Each rabbit was individually housed in a stainless-steel cage and fed with commercial rabbit pellets (Penternakan Hong Lee Sdn. Bhd., Malaysia). Also, freshwater was given ad libitum. The rabbits were acclimatised for one week before the induction of OA. The experimental protocol of the animal study was approved by the Institutional Animal Care and Use Committee (IACUC), Universiti Putra Malaysia (UPM/IACUC/AUP-R034). The rabbits were randomly divided into a control group (*n* = 5), surgically induced group (*n* = 15) and chemically induced group (*n* = 15). Furthermore, the surgically and chemically induced rabbits were divided into three groups based on different time points: week 4, week 8, and week 12. OA was induced at the right knees of the animals.

### Preparation of animal model of osteoarthritis

#### Surgically induced model

In the surgically induced group, OA induction was performed by anterior cruciate ligament transection (ACLT) at the right knee. The animals were anaesthetised with Zoletil® (Virbac, Australia) at 3 mg/kg via intramuscular route and maintained with isofluorane (3%) (Piramal Healthcare, India). The right stifle was prepared in a surgically sterile procedure. In order to achieve optimum visualisation of the anterior cruciate ligament, the knee was placed in complete flexion and the patella was medially dislocated by lateral parapatellar arthrotomy. The ACL was visualised and transected, and the joint capsule and subcutaneous tissue were closed with 4–0 polydioxanone suture upon irrigation with sterile saline. Thereafter, a 3–0 nylon surgical suture was used to close the skin. Tramadol (Duopharma, Malaysia) was given as an analgesics at 2 mg/kg every 12 h for 3 days.

#### Chemically induced model

Monosodium iodoacetate (MIA) (Sigma-Aldrich, USA) was dissolved to a concentration of 25 mg/ml in saline. MIA was injected into the intraarticular space of the stifle joint at 8 mg per joint per rabbit with a volume of 0.32 ml. The induction procedure was performed, while the rabbits were under general anaesthesia using Zoletil® (Virbac, Australia) at 2 mg/kg intramuscularly.

### Sample collection

The rabbits were visually inspected daily for any clinical signs such as weight loss or immobility. At the end of week 4, week 8 and week 12, the rabbits from each group were euthanised using pentobarbital sodium (Vetoquinol, France) at 120 mg/kg intravenously. The knee joints were harvested and immediately fixed in 10% buffered formalin (Sigma-Aldrich, USA). After 24 h, the muscle and tissue surrounding the femur and tibia were removed carefully to avoid damaging the cartilage surface. The proximal femur and distal tibia were placed in 10% buffered formalin.

### Micro-computed tomography evaluation

The distal part of each femur was scanned with a micro-CT scanner (Skyscan, Belgium) using a pixel size of 18 μm. Also, the proximal part of each tibia of the stifle joint was scanned in a similar manner. The current used was 110 μA with a 0.5 mm aluminium filter, and the X-ray tube voltage used was 70 kV. The exposure time was 500 ms, and X-ray projections were obtained at 0.9 degrees intervals with a scanning angular range of 360 degrees. The data were then reconstructed with Skyscan NRecon software (Skyscan, Belgium). Two-dimensional (2D) images were qualitatively evaluated by using DataViewer software (Skyscan, Belgium) in both dorsal and sagittal planes for morphological changes that occurred in different experimental groups. The lining of the joint and the presence or absence of osteophytes in the reconstructed dataset were identified and described.

Micro-CT images were analysed quantitatively by using Skyscan CT-Analyser Software (Skyscan, Luxembourg, Belgium). A stack of regions of interest (ROI) within a volume of interest (VOI) was selected from distal femoral and proximal tibia subchondral bone at the epiphyseal with a semiautomatic contouring method. The parameters of subchondral bone micro-architecture that were analysed included the ratio of bone volume over tissue volume (BV/TV; %), the bone surface-to-volume ratio (BS/BV; mm^2^/mm^3^), trabecular thickness (Tb.Th; mm), trabecular spacing (Tb.Sp; mm) and total porosity (PO; %).

### Histology

The bones from the harvested joint were immediately fixed in 10% buffered formalin (Sigma-Aldrich, USA) and decalcified with 10% formic acid (Nacalai Tesque, Japan). The 10% formic acid was changed every 2 days for 10 days.

#### Embedding and sectioning

The tissue surrounding the joint was carefully removed before embedding. Then, the femur and tibia bone were cut at a dorsal plane. The bones were placed in cassettes and labelled. The tissue processing was done using Leica TP1020 Semi-enclosed Benchtop Tissue Processor (Leica Biosystems, Germany). Next, the cassettes were soaked in 80% alcohol and 95% alcohol for two hours each, followed by 100% alcohol for three hours, chloroform solution for 3 h and lastly in paraffin for 5 h and 30 min.

Then, the bones were embedded in paraffin using Leica EG1150H and EG11559 Modular Tissue Embedding Center (Leica Biosystems, Germany). The paraffin block was sliced using the Reichert-Jung 2045 Multicut Rotary Microtome (Leica Biosystems, Germany) at 5 μm thickness and mounted on a glass slide. The glass slides were dried on the slide warmer overnight. The slides were then deparaffinised and then hydrated with distilled water. They were stained using Weigert's iron haematoxylin working solution for 10 min and washed in running tap water for 10 min. Thereafter, they were stained using Fast green (FCF) solution for 5 min, rinsed rapidly with 1% acetic acid not exceeding 10–15 s and stained using 0.1% Safranin-O solution for 5 min. The slides were dehydrated and cleared with 95% ethyl alcohol and absolute ethyl alcohol, alternately at 2 min each for two times. Lastly, the slides were mounted with a resinous medium to make them not detachable.

#### Histological evaluation

The slides were observed under a microscope (Motic, China) under 10× and 40× magnification and scored independently by two blinded observers. For each sample, two slides were prepared and graded five times at different sections along with the articular cartilage. The changes of articular cartilage were scored using OARSI Scoring System [16].

### Statistical analysis

Micro-architecture of bone parameters for surgically and chemically induced groups was compared using one-factor analysis of variance (ANOVA), and a Tukey's HSD multiple comparison post hoc test was used to classify significant differences between groups. The data that were not normally distributed were tabulated in median form, and statistical comparisons for scoring between groups were made using the Kruskal–Wallis (K–W) nonparametric ANOVA and Dunn's multiple comparison tests. A *p*-value less than 0.05 was considered for a significant difference between groups. Micro-architecture bone parameters and histological evaluation statistical analysis were done using Graphpad Prism 7 (Graphpad Software, USA). Measurement consistency between different examiners were calculated using intraclass correlation coefficient (ICC) using two-way mixed effects model with absolute agreement. Intraclass correlation coefficient (ICC) analysis was performed using IBM SPSS Statistics 23 (SPSS Inc., USA), and an ICC > 0.7 is commonly used to indicate sufficient reliability.

## Results

### General condition of rabbit models

Both groups showed no clinical signs of pain, immobility or reduction in body weight and were generally healthy. The average body weight (range) of the rabbits was 2.4 kg (1.2–3.6 kg) during euthanasia. By physical examination, the surgically induced group developed joint effusion accompanied by joint swelling and warmth which gradually increased over time.

### Two-dimensional micro-CT images assessment

**Fig. 1:**
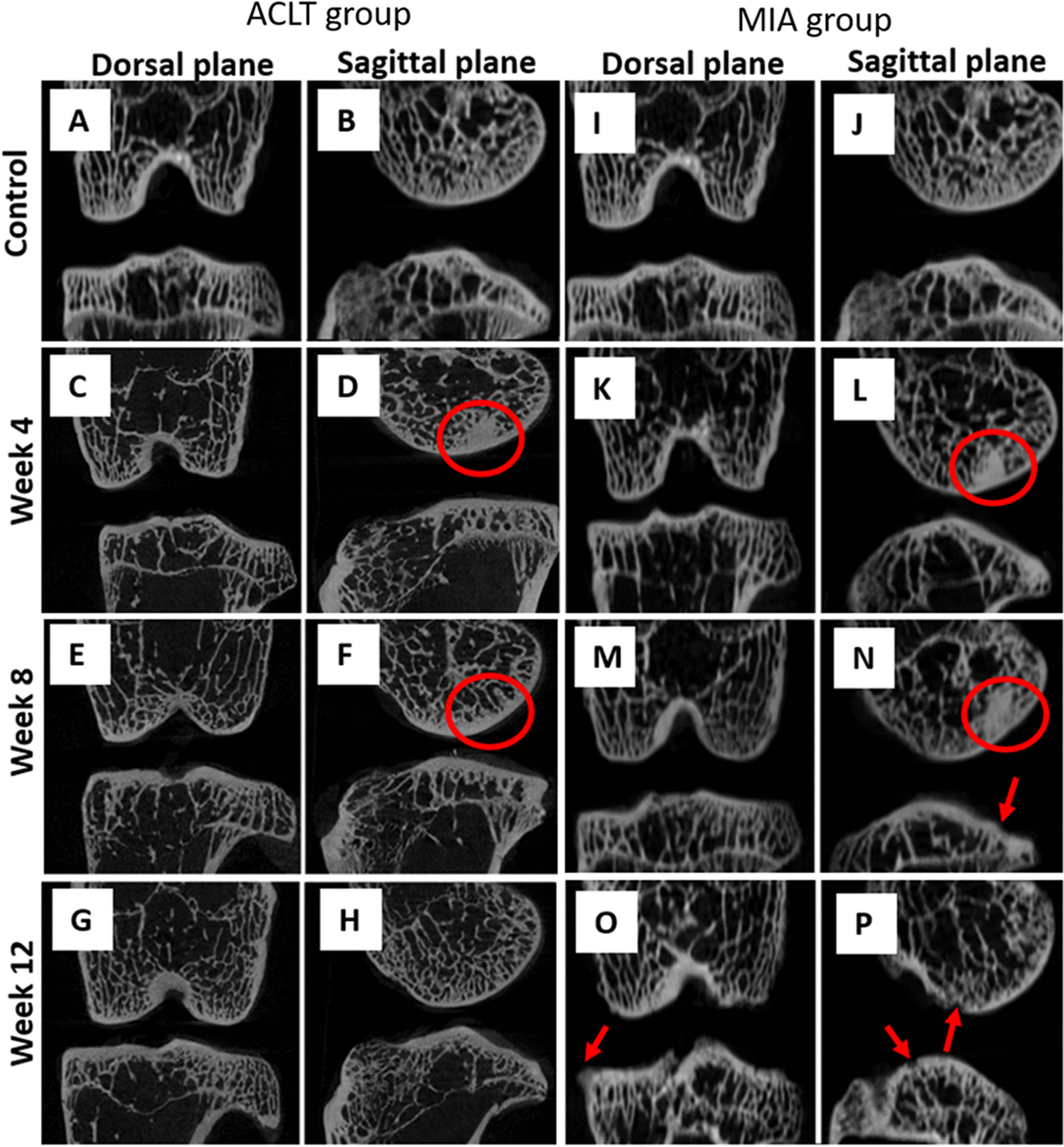
Two-dimensional micro-CT scans of dorsal and sagittal planes of the surgically and chemically induced group. Surgically induced group with the presence of sclerosis at week 4 and week 8 group (red circle). Chemically induced group showed sclerosis in the week 4 and week 8 group (red circle), surface irregularities and osteophytes formation in the week 8 and week 12 group (red arrow)

The micro-CT scans focused on two dorsal and sagittal planes (Fig. 1). The control group showed normal subchondral bone and metaphyseal region with a regular and smooth contour. In contrast, the surgically induced group revealed the presence of sclerosis at week 4 (Fig. 1D) and week 8 group (Fig. 1F). In the chemically induced group, there were surface irregularities in the subchondral region of the femur and tibia with marginal sclerosis at week 4 (Fig. 1L) and week 8 groups (Fig. 1N). Osteophytes formation was also observed in the week 12 group (Fig. 1O, P).

### Quantitative bone micro-architecture assessment

Based on the bone micro-architecture assessment, the parameters extracted included BV/TV, BS/BV, Tb.Th, Tb.Sp and PO. The mean and standard deviation of each parameter for the surgically and chemically induced groups are presented in Tables 1 and 2, respectively.

**Table 1:** Micro-architecture parameters of the subchondral bone of right stifle joints of femur obtained from surgically and chemically induced OA in rabbit model

	BV/TV (%)		BS/BV (mm^2^/mm^3^)		Tb.Th (mm)		Tb.Sp (mm)		PO (%)	
	Mean ± SD		Mean ± SD		Mean ± SD		Mean ± SD		Mean ± SD	
	ACLT	MIA	ACLT	MIA	ACLT	MIA	ACLT	MIA	ACLT	MIA
Control	35.68 ± 2.89	35.68 ± 2.89	18.09 ± 1.41	18.09 ± 1.41	0.18 ± 0.01	0.18 ± 0.01	0.50 ± 0.05	0.50 ± 0.05	64.32 ± 2.89	64.32 ± 2.89
Week 4	35.31 ± 3.16	31.57 ± 6.03	16.99 ± 0.92	19.55 ± 1.89	0.19 ± 0.01	0.17 ± 0.01	0.54 ± 0.07	0.54 ± 0.12	64.69 ± 3.17	68.43 ± 6.03
Week 8	32.80 ± 5.43	30.89 ± 2.55	19.55 ± 1.18	18.38 ± 1.39	0.17 ± 0.01	0.18 ± 0.01	0.50 ± 0.09	0.59 ± 0.09	67.2 ± 5.43	69.11 ± 2.55
Week 12	35.24 ± 2.22	27.12 ± 4.40	18.55 ± 1.34	19.63 ± 1.32	0.18 ± 0.01	0.18 ± 0.01	0.50 ± 0.10	0.65 ± 0.18	64.76 ± 2.22	72.88 ± 4.40

**Table 2:** Micro-architecture parameters of the subchondral bone of right stifle joints of tibia obtained from surgically and chemically induced OA in rabbit model

	BV/TV (%)		BS/BV (mm^2^/mm^3^)		Tb.Th (mm)		Tb.Sp (mm)		PO (%)	
	Mean ± SD		Mean ± SD		Mean ± SD		Mean ± SD		Mean ± SD	
	ACLT	MIA	ACLT	MIA	ACLT	MIA	ACLT	MIA	ACLT	MIA
Control	34.90 ± 5.04	34.90 ± 5.04	20.08 ± 1.74	20.08 ± 1.74	0.18 ± 0.01	0.18 ± 0.01	0.53 ± 0.05	0.53 ± 0.05	65.10 ± 5.04	65.10 ± 5.04
Week 4	33.96 ± 3.39	30.88 ± 6.56	19.68 ± 1.27	21.58 ± 2.03	0.18 ± 0.01	0.17 ± 0.01	0.53 ± 0.08	0.54 ± 0.11	72.32 ± 24.06	69.12 ± 6.57
Week 8	32.26 ± 5.36	30.18 ± 1.91	21.22 ± 1.40	20.74 ± 1.41	0.17 ± 0.01	0.18 ± 0.01	0.54 ± 0.10	0.60 ± 0.07	67.74 ± 5.36	69.82 ± 1.91
Week 12	32.82 ± 1.99	26.82 ± 3.50	20.76 ± 0.65	22.02 ± 1.13	0.17 ± 0.01	0.17 ± 0.01	0.51 ± 0.04	0.61 ± 0.14	67.18 ± 1.99	73.18 ± 3.50

**Fig. 2:**
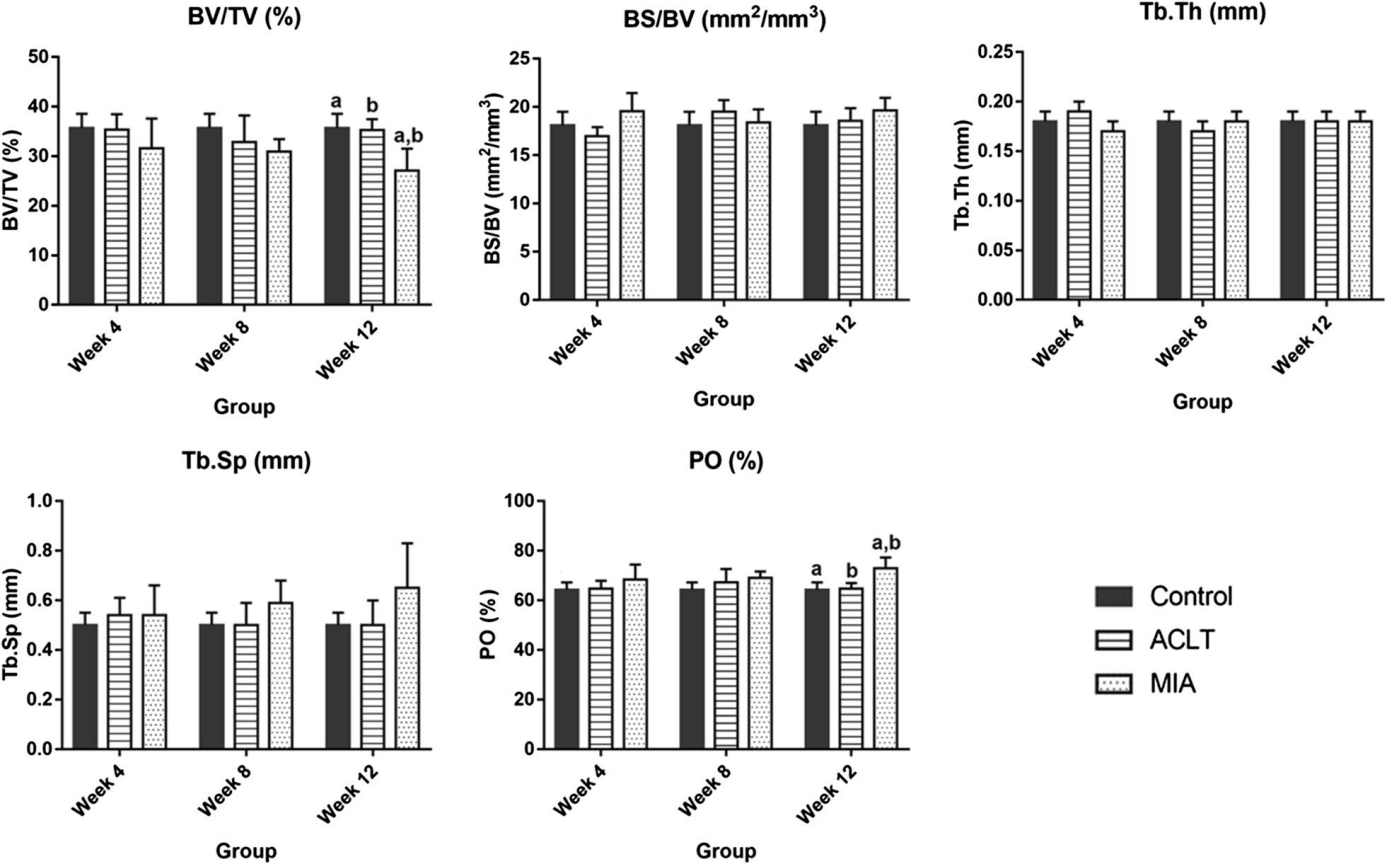
Comparison of the bone micro-architecture of the subchondral bone of right stifle joints of the femur between different types of OA induction for control, week 4, week 8 and week 12 groups. The parameters analysed include **a** BV/TV, **b** BS/BV, **c** Tb.Th, **d** Tb.Sp and **e** PO. Results are presented as mean ± SD. *BV/TV* bone-to-tissue volume ratio, *BS/BV* bone surface-to-volume ratio, *Tb.Th* trabecular thickness, *Tb.Sp* trabecular spacing, *PO* total porosity. Values with same superscript letters (across the column) are significantly different (*p* < 0.05)

As shown in Fig. 2, for parameters regarding femur bone, BV/TV for the surgically induced group decreased at week 8 and increased at week 12, whereas BV/TV value for the chemically induced group decreased from week 4 until week 12. The BV/TV value for the chemically induced group decreased significantly at week 12 compared to the control and surgically induced group. BS/BV value for the surgically induced group decreased at week 4, increased at week 8 and slightly decreased at week 12. Inconsistent findings were observed in the chemically induced group where the BS/BV value increased at week 4, decreased at week 8 and had the highest value at week 12. Also, BS/BV value for the chemically induced group exhibited an increase as compared to the surgically induced group at week 4.

Tb.Th increased at week 4 in the surgically induced group, followed by a decrease at week 8 and elevated again at week 12. On the other hand, Tb.Th value for the chemically induced group decreased at week 4, elevated at week 8 and remained constant at week 12. For the surgically induced group, Tb.Sp value showed an increase at week 4, declined at week 8 and maintained at week 12. For the chemically induced group, Tb.Sp value recorded a persistent increase from week 4 until week 12. The PO value for the surgically induced group increased at week 4 and week 8 followed by a decrease at week 12, whereas the values consistently increased in the chemically induced group from week 4 to week 12. There was a significant difference in the PO value between the chemically induced group, control and surgically induced group at week 12.

**Fig. 3:**
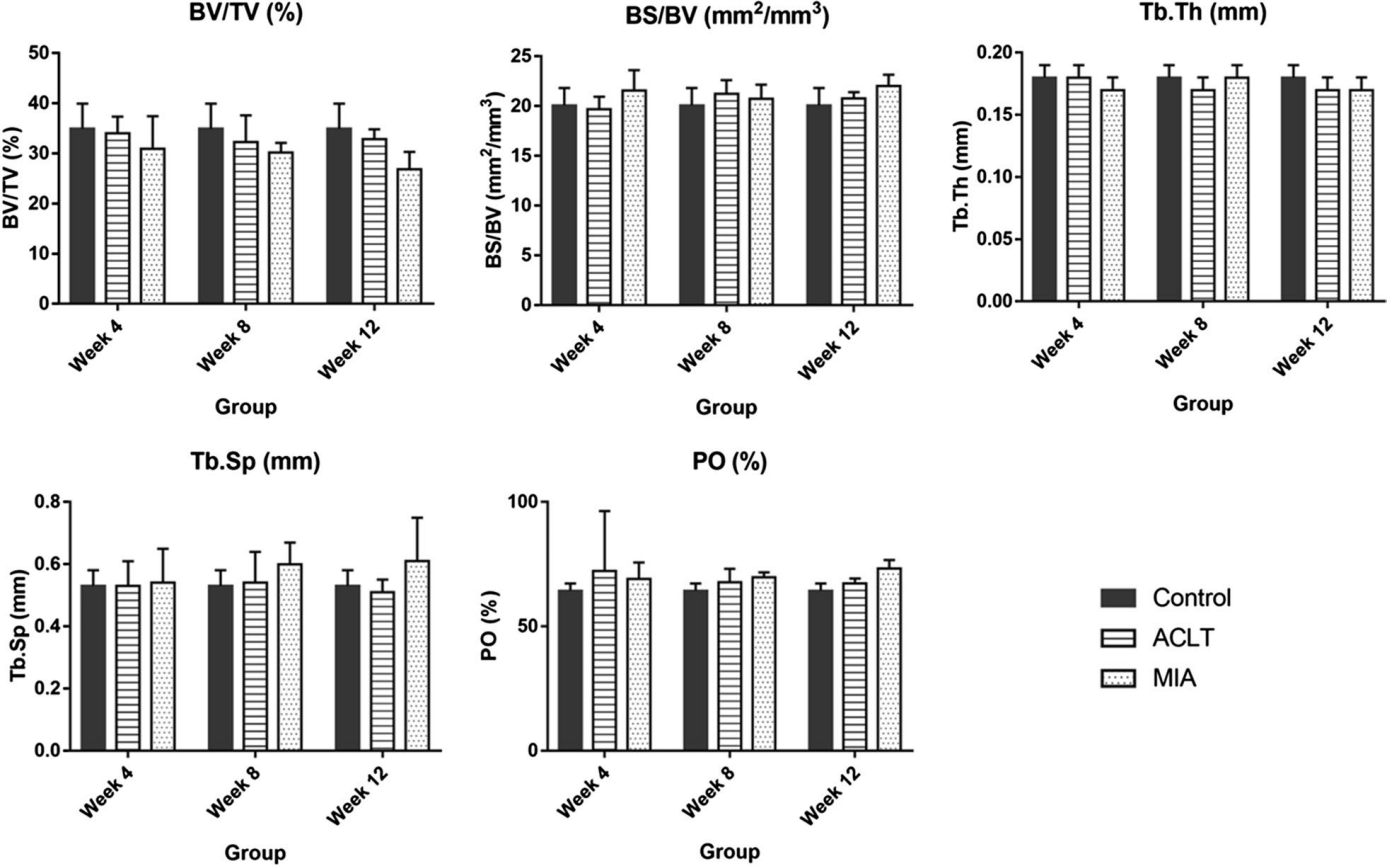
Comparison of the bone micro-architecture of the subchondral bone of right stifle joints of the tibia between different types of OA induction for control, week 4, week 8 and week 12 groups. The parameters analysed include **a** BV/TV, **b** BS/BV, **c** Tb.Th, **d** Tb.Sp and **e** PO. Results were presented as mean ± SD. *BV/TV* bone-to-tissue volume ratio, *BS/BV* bone surface-to-volume ratio, *Tb.Th* trabecular thickness, *Tb.Sp* trabecular spacing, *PO* total porosity

For parameters regarding the tibia bone, BV/TV value for the surgically induced group initially decreased at weeks 4 and 8 but increased slightly at week 12 (Fig. 3). In contrast, BV/TV value continually decreased from week 4 until week 12 in the chemically induced group. BS/BV value decreased at week 4, increased at week 8 and decreased at week 12 in the surgically induced group.

Tb.Th value for the surgically induced group decreased during week 8 and recorded the same value at week 12. On the other hand, the chemically induced group recorded a decrease in Tb.Th value at week 4, elevated at week 8 and decreased at week 12 before reverting to the initial value (i.e. week 4). Tb.Sp value for the surgically induced group increased at week 4, maintained at week 8 and decreased at week 12. For the chemically induced group, Tb.Sp value increased gradually from week 4 until week 12. The PO value for the surgically induced group increased at week 4 and decreased at week 8 and week 12. For the chemically induced group, PO value increased continuously from week 4 until week 12.

### Histopathological grading and statistical analysis

**Fig. 4:**
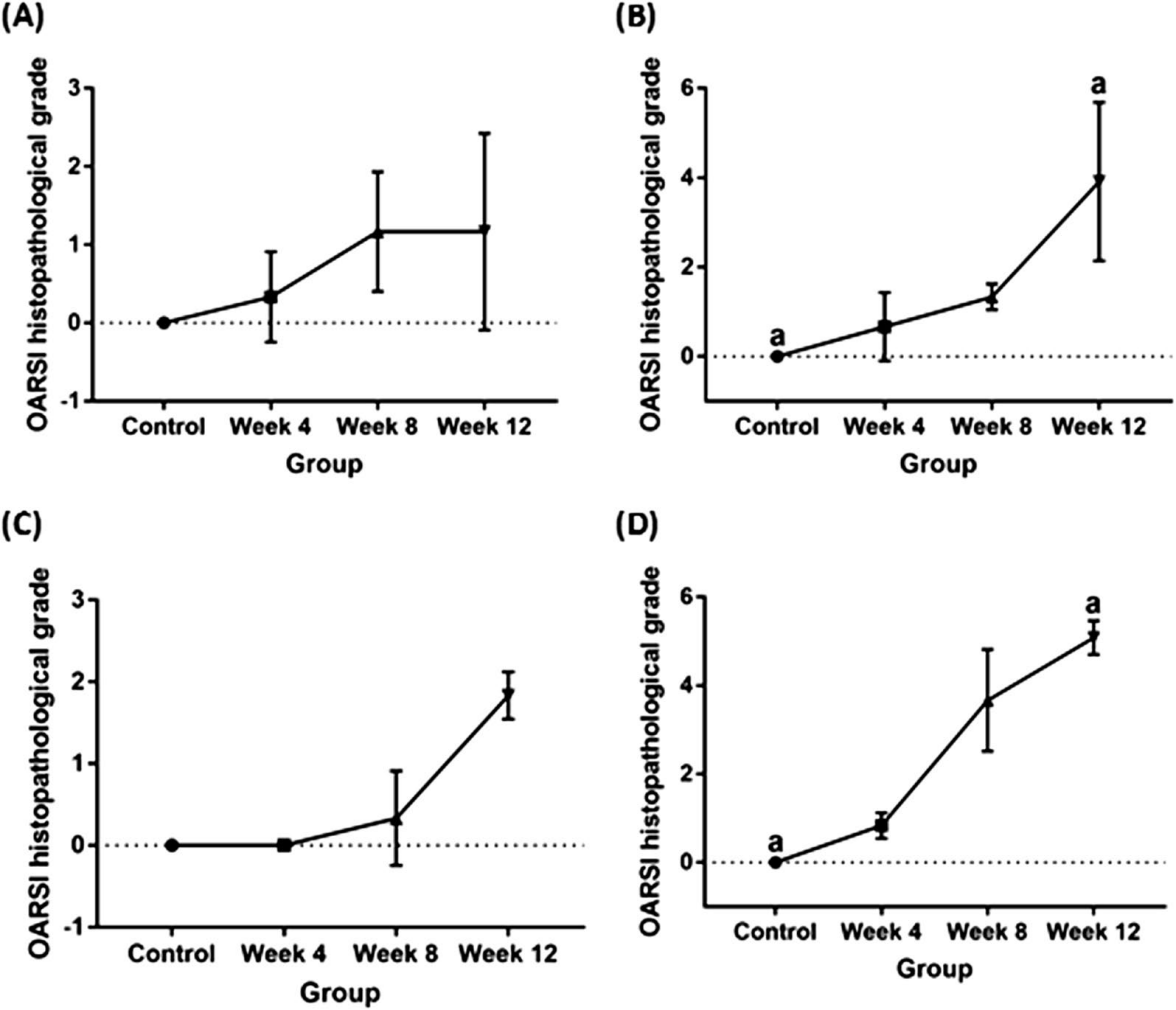
OARSI histological scores for **A** surgically induced femoral cartilage, **B** chemically induced femoral cartilage, **C** surgically induced tibial cartilage, **D** chemically induced tibial cartilage. Values are presented in median with 95% confidence interval (*n* = 5). There was a significant difference (Dunn's multiple comparisons, *p* < 0.05) between points with the same letter within the groups

**Fig. 5:**
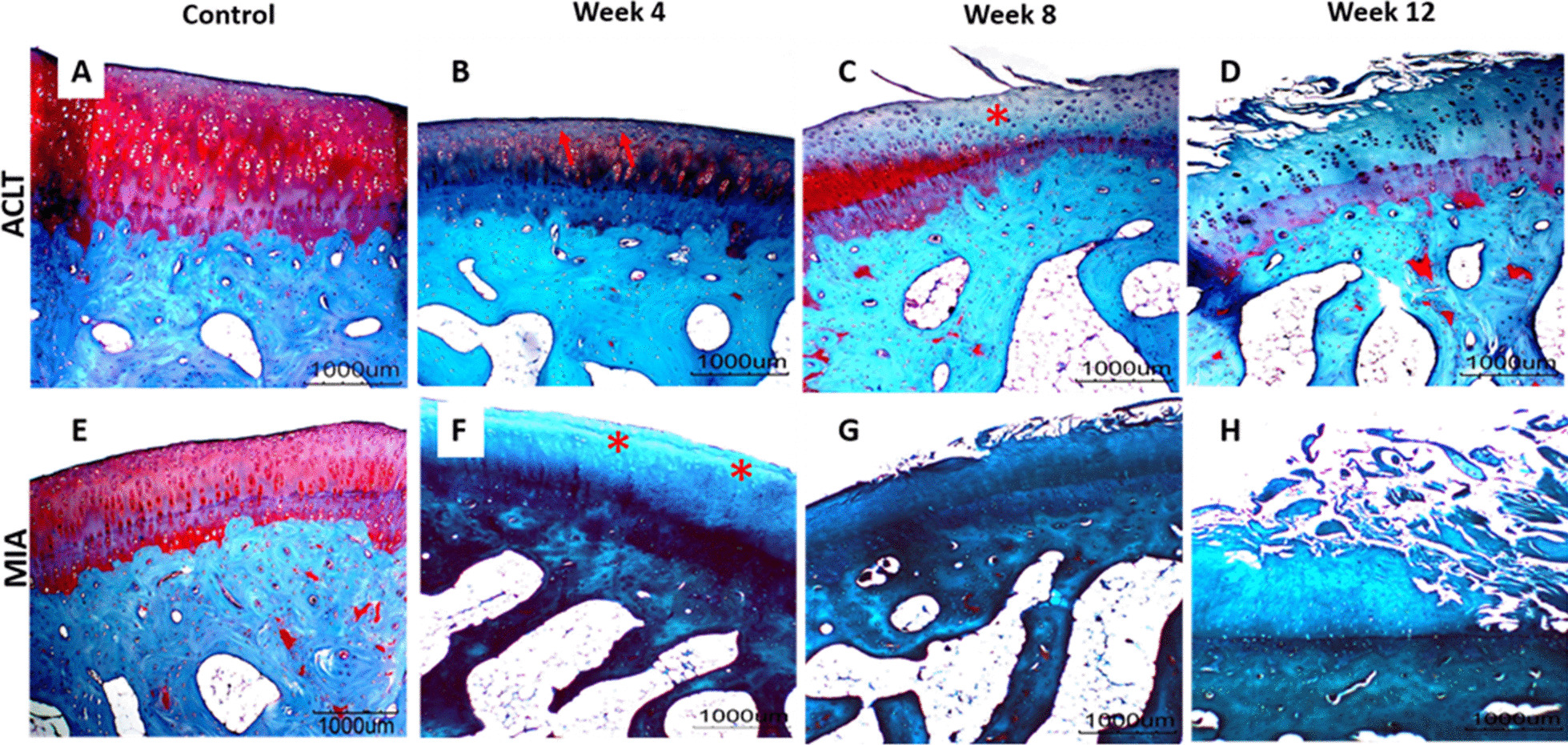
Representative histology images of femoral articular cartilage. **A–D** surgically induced group. **E–H** chemically induced group. Safranin O/fast green stained with 20× magnification. At week 4, the surgically induced group showed apoptosis indicated by empty chondrons (red arrow) and superficial fibrillation at week 8 (asterisk). Uneven cartilage surface can be seen at week 4 in the chemically induced group (asterisk) and cartilage erosion at week 12

**Fig. 6:**
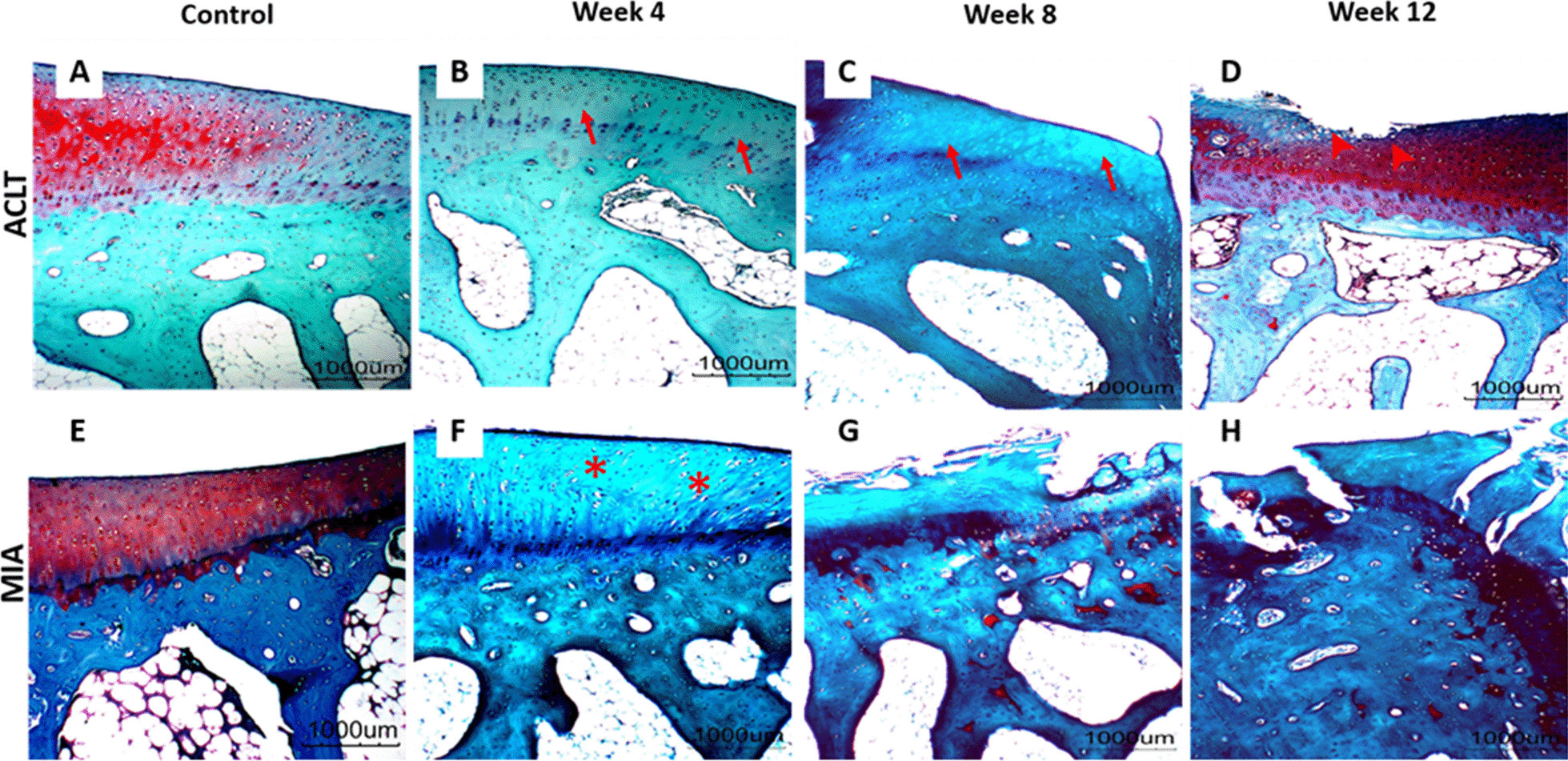
Representative histology images of tibial articular cartilage. **A–D** surgically induced group. **E–H** chemically induced group. Safranin O/fast green stained with 20× magnification. Apoptosis indicated by empty chondrons at week 4 and week 8 in the surgically induced group (red arrow). Superficial and upper mid-zone erosion can be seen at week 12 in the surgically induce group (arrowhead). For the chemically induced group, a lack of chondrocytes can be seen at week 4 (asterisk) and complete erosion at week 12

The histopathological grading of the articular linings of femur and tibia of all the experimental groups is summarised in Table 3, Figs. 4, 5 and 6. The control group was graded 0 with normal chondrocytes distribution and smooth cartilage surface. In the surgically induced group, the articular cartilage was graded with a median of 0 at week 4 (range 0–1), 1 at week 8 (range 0.5–2) and 1 at Week 12 (range 0–2.5). At week 4 and week 8 group, the superficial zones of the articular cartilage were still intact. However, uneven articular surface and superficial fibrillation were observed (Fig. 5B, C). At week 12 (Fig. 5D), surface discontinuity indicated by superficial zone fibrillation was observed.

**Table 3:** OARSI histopathological grade of femur and tibia in the control, and at weeks 4, 8 and 12 for the surgically induced and chemically induced group

	Control		Week 4		Week 8		Week 12	
	ACLT (median, range)	MIA (median, range)	ACLT (median, range)	MIA (median, range)	ACLT (median, range)	MIA (median, range)	ACLT (median, range)	MIA (median, range)
Femur	0	0	0 (0–1)	0.5 (0–1.5)	1 (0.5–2)	1.5 (1–1.5)	1 (0–2.5)	4.25 (2–5.5)
Tibia	0	0	0 (0)	1 (0.5–1)	0 (0–1)	3 (3–5)	2 (1.5–2.0)	5 (4.5–5.5)

For the chemically induced group, the articular cartilage at week 4 recorded a median of 0.5 (range 0–1.5), 1.5 at week 8 (range 1–1.5) and 4.5 at week 12 group (range 2–5.5). The median score at week 12 was significantly different compared with the control group (*p* = 0.0184). In addition, the chemically induced group showed more severe articular cartilage changes compared with the surgically induced group in which cartilage erosion was observed at week 12 (Fig. 5H).

For the tibia bone, the surgically induced group recorded a median score of 0 at week 4 and week 8 (range 0–1) and 2 (range 1.5–2) at week 12. At week 4, the samples graded as 0 were characterised by a normal morphology of cartilage surface. However, at week 8, apoptosis characterised with empty chondrons and superficial fibrillation was observed. At week 12, there were surface discontinuity and deep fibrillation through the superficial zone (Fig. 6D).

In the chemically induced group, the articular cartilage of the tibia at weeks 4, 8 and 12 was graded as 1 (range 0.5–1) and 3 (range 3–5) and 5 (range 4.5–5.5), respectively. At week 12, there was a significant difference between the median scores (i.e. articular cartilage of the tibia) between the chemically induced group and the control (*p* = 0.0208). The intraobserver reliability comparison was high. The ICC for femur in surgically induced group was 0.923 (95% CI 0.765–0.977), and chemically induced group was 0.907 (95% CI 0.720–0.972). The ICC for tibia was 0.966 (95% CI 0.888–0.990) in surgically induced group and 0.980 (95% CI 0.935–0.994) in chemically induced group. The *p*-value for all statistical analysis were provided in Additional file 1.

## Discussion

This study characterised femoral and tibial subchondral bone micro-architecture and articular cartilage changes of two widely used OA induction methods in a rabbit model using micro-CT and histological studies. The strength of this study involved the quantitative examination of temporal changes of subchondral bone and articular cartilage using micro-CT and histological scoring for different induction methods.

In this study, there was a development of joint effusion in the surgically induced group based on visual inspection from week 4 to week 12 with a substantial increase until week 12. This finding is consistent with the reports by Wachsmuth et al. [17] in which severe joint effusion was observed two weeks after surgery and persisted until week 8 in an in vivo rabbit model of OA. The surgically induced group had a substantially higher joint effusion than the chemically induced group. Since a more invasive ACL section was performed in the surgically induced group, the pronounced joint effusion may be attributed to higher severity of post-operative inflammation [18]. Nevertheless, the joint effusion and swelling had no significant effect on the rabbits' appetite and movement as the bodyweight increased continuously and no sign of immobilisation was detected.

Two-dimensional morphological findings revealed sclerosis in both surgically and chemically induced groups at week 4 and week 8. Subchondral bone sclerosis is considered an important attribute of OA initiation and progression. The subchondral bone acts as a shock absorber, distributes forces and adapts to maintain joint conformation and prevent stress concentration [19]. Radin and Rose [20] postulated OA might begin with subchondral bone adaptation by local stiffening to generate a steep stiffness gradient and induces high shear forces in the cartilage.

There were joint surface irregularities and osteophytes formation at weeks 8 and 12 in the chemically induced group. Osteophytosis is regulated by several growth factors and cytokines such as TGF-β secreted by chondrocytes, which is released following a joint tissue injury. This event might be caused by chondrocytes alteration after MIA administration, thus stimulating the formation of an aberrant bone via nestin-positive mesenchymal stem cells or osteoprogenitor cells [21]. Although some osteoarthritic changes were seen in two-dimensional images, three-dimensional imaging techniques using micro-CT enable the morphological changes in bone to accurately quantified. Therefore, several bone parameters were measured in this study.

The proportion of bone volume in total tissue volume is represented as BV/TV, which is a measure of changes in total bone volume [22]. In the surgically induced group, the values of BV/TV decreased at weeks 4 and 8 and increased at week 12 in both femur and tibia. The result suggested that trabecular bone formation was already taking place in the epiphyseal region at week 12. A previous study conducted in surgically induced dogs reported similar findings as high bone remodelling occurred during the latter weeks [23]. The increase in BV/TV may represent an advanced OA as late-stage OA is dominated by bone formation and higher osteoblastic activities [24]. For the chemically induced group, the BV/TV value in the femur and tibia decreased initially after induction and persisted until week 12. The continuous decline of BV/TV value may be due to less loading of the injected knee as demonstrated in a previous study in which the injection of MIA into rats’ knee joint triggered weight distribution changes in the hind paw, which resulted in bone loss [9].

BS/BV is the ratio of the selected bone surface to the bone volume and depicts the degree of bone turnover [22]. In this study, the BS/BV values were inversely related to Tb.Th. The negative correlation between BS/BV and Tb.Th may imply that bone structure complexity affects trabeculae thickness and vice versa. For the surgically induced group, BS/BV values decreased at week 4, increased at week 8 and decreased at week 12. In contrast, the chemically induced group showed contradicting and insignificant outcomes in both the femur and tibia.

Tb.Th is a measure of trabecular bone thickness, and the value is directly proportional to the degree of bone formation. In the surgically induced group, Tb.Th values increased in the femur at weeks 4 and 12 and decreased in the femur and tibia at week 8. This may be attributed to knee instability resulting from irregular anterior–posterior and rotational movements after ACL transection [25]. Modified contact pressures, stress distributions and reduced muscle activity are linked to altered loading conditions in ACLT joints [26]. As a result, an adaptive bone remodelling mechanism ensues, which may result in the elevation of Tb.Th. In addition, increased Tb.Th values may arise due to imbalanced bone resorption and forming process in the less loaded joint compartment [25] at week 4. This is also supported by a decrease in the femoral Tb.Sp value in the surgically induced group at week 8 and tibial Tb.Sp value at week 12. The average distance between trabecular bones is indicated by Tb.Sp, and thus, the smaller the separation, the higher the bone density. Due to reduced mechanical properties caused by instability after surgical induction, subchondral osteoblast may enhance anabolic activity via IL-6 which promotes osteoblast differentiation by stimulating the synthesis of IGF-1 and IGF binding protein, leading to increased bone formation [27]. This process culminates in a narrowed Tb.Sp to bear abnormal joint stress [28].

In the chemically induced group, it is suggested that bone resorption persisted until week 8, indicated by increased bone turnover represented by a decrease in Tb.Th value at week 4. Also, Tb.Sp increased consistently from week 4 until week 12 in the femur and tibia. Damage to the chondrocyte caused by MIA may result in increased mechanical loading leading to micro-cracks. This damage stimulates the osteocytes in the damaged area to generate RANKL (receptor activator of nuclear factor B ligand), also known as TNF superfamily member 11 [TNFSF11]), and downregulate osteoprotegerin (OPG), an inactivating receptor for RANKL, thereby stimulating bone resorption [29, 30]. In animal models of OA, a lower OPG/RANKL ratio has been observed [31], while RANKL and its isoforms were reported to be differentially expressed in subchondral bone osteoblasts from patients with OA in humans [32]. This suggests that bone changes differed between the surgically and chemically induced groups considering the Tb.Th and Tb.Sp results.

PO is defined as total porosity which is the volume of all open plus closed pores as per cent of the total volume [33]*.* Surgically induced group recorded an initial increase in PO value, followed by a decrease at week 12 in femur, whereas the decrease in PO started at week 8 and persisted until week 12 for tibia. This result corroborates the trends with PTOA [34]. The decrease in PO may be due to the physical response to repeated impulsive loads applied over time [35]. The chemically induced group showed increased PO from week 4 until week 12 in femur and tibia, and this is in line with previous studies [36, 37]. The increase in osteoclastic activity and trabecular bone resorption in early OA may be caused by increased local blood flow induced by angiogenic factors [38] as well as enhanced bone–cartilage crosstalk, which causes the elevation of PO in subchondral bone plate [39, 40].

Minimal changes in the articular cartilage were observed in the surgically induced group based on the histological scoring throughout all time points. Surgical induction will cause joint instability leading to excessive mechanical loading in the injured joint articular cartilage. Excessive mechanical loading induces gremlin-1, which in turn activates NF-κB signalling. These events enhance the release of catabolic enzymes such as matrix metalloproteinase 13 (MMP13), disintegrin and metalloproteinase with thrombospondin motifs 5 (ADAMTS5) and subsequently cause articular cartilage degeneration [41]. However, superficial cartilage fibrillation observed at week 4 in the tibia and week 8 in the femur indicates initial degenerative changes in OA articular cartilage that may be pronounced over time [42].

As opposed to the surgically induced group, the chemically induced group showed more severe changes in articular cartilage based on the higher histological scoring. Mild changes were observed at week 4 and week 12, with a pre-existing erosion at the articular cartilage of the femur and tibia. The changes in the matrix and chondrocytes were prominent in the chemically induced group, where extensive cartilage damage characterised by fibrillation, fragmentation, ulceration and cluster formation was observed.

Thus, it can be speculated that the surgically induced group showed changes in bone arising from the changes in the articular cartilage. This was demonstrated by bone remodelling which occurred at week 12 following late-stage OA. However, articular cartilage scoring revealed minimal changes. Bone injury after surgical induction is followed by chondrocytes response leading to either modulating synthetic action or increasing the level of catabolic enzymes. These processes result in the breakdown of the cartilage.

On the other hand, in the chemically induced group, changes in articular cartilage precede changes in the bone, following a chemical induction in which glyceraldehyde-3-phosphate precipitates chondrocytes death and subsequent breakdown of cartilage matrix [43]. Thus, articular cartilage will attempt to self-repair by reducing excessive mechanical loads on the adjacent subchondral bone. The ratio of receptor activator of nuclear factor B ligand (RANKL)/osteoprotegerin (OPG) expression in osteocytes increases as a result of underloading, resulting in excessive osteoclastogenesis and increased bone resorption activity [44, 45].

Chemical induction using MIA inhibits glyceraldehyde-3-phosphate dehydrogenase which induces the production of reactive oxygen species (ROS) and caspase activation, leading to chondrocyte death—a condition similar to primary OA [46]. The death of chondrocytes causes articular cartilage thinning and lack of cellularity, presence of osteophytes and separation of the articular cartilage from the subchondral bone [10].

Overall, although this study is limited by the small numbers of animal in each group, this study provides significant preliminary data regarding temporal OA progression for different induction methods which may justify larger scale studies to be conducted in the future.

## Conclusion

In this study, it is suggested that subchondral bone remodelling preceded articular cartilage damage in the ACLT group and vice versa in the MIA group. The result may reveal different pathogenic mechanisms for both induction methods, therefore providing more insights for targeted therapeutic strategies, monitoring disease progression and treatment outcome.

## Supplementary Information

**Additional file 1: Supplementary p-values.** *p*-values for micro-CT parameters and histological scoring statistical analysis.

## Data Availability

The data underlying this article will be shared upon reasonable request to the corresponding author.

## Abbreviations

ADAMTS5: A disintegrin and metalloproteinase with thrombospondin motifs 5
ACL: Anterior cruciate ligament
ACLT: Anterior cruciate ligament transection
ANOVA: Analysis of variance
BS/BV: Bone surface-to-volume ratio
BV/TV: Bone volume over tissue volume
IACUC: Institutional Animal Care and Use Committee
IGF: Insulin-like growth factor
IGF-1: Insulin-like growth factor 1
MIA: Monosodium iodoacetate
MMP13: Matrix metalloproteinase 13
NF-κB: Nuclear factor kappa B
OA: Osteoarthritis
OARSI: Osteoarthritis Research Society International
OPG: Osteoprotegerin
PO: Total porosity
PTOA: Post-traumatic osteoarthritis
RANKL: Receptor activator of nuclear factor kappa-Β ligand
ROS: Reactive oxygen species
Tb.Sp: Trabecular spacing
Tb.Th: Trabecular thickness
TGF-β: Transforming growth factor alpha
